# Valued Officials

**Published:** 1890-08-23

**Authors:** 


### Passing Topics.

### VALUED OFFICIALS.

The officers of the London Hospital have no excuse for a lack of *esprit*. They are vouchsafed that which persons of integrity and sensibility placed in positions of trust must ever regard as the very highest incentives to zeal—the full and complete confidence of those to whom they are primarily responsible. However divergent the views taken of the evidence tendered by the several witnesses before the Lords' Committee in respect of the London Hospital—whether we are inclined to attach sinister importance to the statements of the complainants, or whether we are persuaded that these are to be received with suspicion as emanating from persons mostly in the position of discharged servants—no generous mind can fail to admire, and some of us will be disposed to envy, the good relations which have been shown to subsist between the committee and its officers.

Doubtless it suits the present purpose of certain people in search for sensation, and not indisposed to play the part of wreckers, to attempt to sow discord by sneering at what these wiseacres are pleased to term the impotence of the committee, but a moment's reflection will convince every sensible man that nothing would more surely reduce a hospital committee to impotence than a want of harmony with its executive, while upon the other hand, it may be added, that the existence of pleasant relations affords reasonable presumption of all-round efficiency. Where there is mutual respect, there must be something to call it forth, and just as a capable committee would demand efficient officers, the latter are not likely to be satisfied with indifferent directors. It is not enough that one portion of the machinery should be perfect. If we would have it to work smoothly, it must be capable as a whole. This is not to assert that the House Committee of the London Hospital is infallible, any more than its officers are. *Humanum est errare* was among the earliest, as alas! it was among the most intelligible traditions of our infantine Latinity, and in this connection we are of opinion that amid other matters calling for comment, it was an error of judgment to relieve the house governor of all responsibility in regard to the nurses—to render the matron, in fact, independent of her nominal chief.

But apart from such criticisms, we consider the cordial expressions of the Treasurer, Chairman of Committee, and others, concerning both the House Governor and Matron, and the resolution of unabated confidence in the latter which the Committee has recently passed, are honourable to all parties alike, and are calculated in the highest degree to conserve and promote the best interests of the institution.

Nor will we consent to believe, at the bidding of some irrational scribes, that the confidence is misplaced. Among all hospital workers, Mr. Nixon's name is regarded with respect and affection, and we hold that there is no testimony more solid and convincing as there is no criticism more searching than that rendered by a man's peers—those who know him not only in his personality but are acquainted with every detail of his official work, and by actual experience of somewhat similar conditions, are enabled to appraise his merits or his demerits with almost mathematical precision.

We congratulate alike the Committee and their staff. Each is loyal to the other, and in that fact we have the best guarantee that the management is capably conducted. That being so, we need not fear the neglect of such reforms as altering circumstances call for, and would continue to call for, though time grew into eternity and not one month was barren of legislation. Of all the persons in this country who wish for real reforms and improvements in hospital management, we are quite sure there are none who desire them more fervently and honestly than the managers and principal officials of the London Hospital. If orders of merit are ever deserved by any class of charitable workers, and they certainly are, the workers at the London are assuredly entitled to receive distinctions of the highest class. Agricola.

